# Four new East Asian species of *Aleurodiscus* with echinulate basidiospores

**DOI:** 10.3897/mycokeys.52.34066

**Published:** 2019-05-09

**Authors:** Sheng-Hua Wu, Chia-Ling Wei, Yu-Ting Lin, Chiung-Chih Chang, Shuang-Hui He

**Affiliations:** 1 Department of Biology, National Museum of Natural Science, Taichung 40419, Taiwan National Museum of Natural Science Taichung Taiwan; 2 Institute of Microbiology, Beijing Forestry University, Beijing 100083, China Beijing Forestry University Beijing China

**Keywords:** China, corticioid fungi, Taiwan, taxonomy, wood-decaying fungi

## Abstract

Four new species of *Aleurodiscus* sensu lato with echinulate basidiospores are described from East Asia: *A.alpinus*, *A.pinicola*, *A.senticosus*, and *A.sichuanensis*. *Aleurodiscusalpinus* is from northwest Yunnan of China where it occurs on *Rhododendron* in montane habitats. *Aleurodiscuspinicola* occurs on *Pinus* in montane settings in Taiwan and northwest Yunnan. *Aleurodiscussenticosus* is from subtropical Taiwan, where it occurs on angiosperms. *Aleurodiscussichuanensis* is reported from southwest China on angiosperms in montane environments. Phylogenetic relationships of these four new species were inferred from analyses of a combined dataset consisting of three genetic markers, viz. 28S, nuc rDNA ITS1-5.8S-ITS2 (ITS), and a portion of the translation elongation factor 1-alpha gene, *TEF1*.

## Introduction

The genus *Aleurodiscus* Rabenh. ex J. Schröt. belongs to the Stereaceae Pilát of the Russulales Kreisel ex P.M. Kirk, P.F. Cannon & J.C. David. However, whether to keep *Aleurodiscus* in a broad or a narrow sense has long been a puzzling issue in the taxonomy of Basidiomycota (Boidin et al 1985; Núñez and Ryvarden 1997; Wu et al. 2001; Larsson and Larsson 2003; Miller et al. 2006; Larsson 2007; Wu et al. 2010), because diagnostic characters are highly variable among species. *Aleurodiscus* s.l. is characterized by cupulate, effused or effused-reflexed basidiocarps, a monomitic or dimitic hyphal system with simple-septate or clamped generative hyphae, smooth or ornamented amyloid basidiospores, and sterile organs such as acanthophyses, gloeocystidia, hyphidia, and dendrohyphidia (Núñez and Ryvarden 1997). The characteristics used for separating segregate genera within *Aleurodiscus* s.l. (*Acanthobasidium* Oberw., *Acanthofungus* Sheng H. Wu et al, *Acanthophysellum* Parmasto, *Aleurobotrys* Boidin, *Aleurodiscus* s.s., *Aleurocystidiellum* P.A. Lemke, *Gloeosoma* Bres., and *Neoaleurodiscus* Sheng H. Wu) as well as *Stereum* Hill ex Pers. and *Xylobolus* P. Karst. were provided by Wu et al. (2001, table 1) and Wu et al. (2010, table 1). Currently, 169 names are recorded under *Aleurodiscus*, of which about 85 taxa are generally accepted worldwide (http://www.indexfungorum.org/). Since the year 2000, new species of *Aleurodiscus* s.l. have been proposed by Simpson and Grgurinovic (2003), Hjortstam et al. (2009), Ryvarden et al. (2012), Gorjón et al. (2013), Maninder et al. (2014), Dai and He (2016), Dai et al. (2017a, b), Ghobad-Nejhad and Langer (2018), and Tian et al. (2018). Since the phylogenetic relationships of the taxa in *Aleurodiscus* s.l., as well as in the Stereaceae at large, are not resolved, we adopt a broad and inclusive generic concept of *Aleurodiscus* for the new taxa presented in this study.

During a two-decade long, ongoing survey of corticioid fungi from mainland China and Taiwan, we have found four new species of *Aleurodiscus* with echinulate basidiospores based on morphological characters. In addition, phylogenetic analyses of a nuclear rDNA 28S D1–D2 domains (28S) dataset and analyses of a second dataset consisting of three genetic markers – nuc rDNA 28S D1–D2 domains (28S), nuc rDNA ITS1-5.8S-ITS2 (ITS), and translation elongation factor 1-alpha (*TEF1*) – are performed to complement our morphological observations and place the newly described species in a molecular phylogenetic framework.

## Materials and methods

### Morphological and cultural studies

Macroscopic and microscopic studies were based on dried specimens. Color names from Rayner (1970) are capitalized. Thin free-hand sections of basidiocarps were prepared for microscopic study. For observations and measurements of microscopic characters, sections were mounted in 5% KOH to ensure rehydration. A blue-black color change with Melzer’s reagent (IKI) indicates an amyloid reaction. Cotton blue (CB) was used as mounting medium to determine cyanophily. Sulphoaldehyde (SA) was used to detect a sulphuric reaction of gloeocystidia; a bluish black color change with SA indicates a positive reaction. The following abbreviations are used for basidiospore measurements: L = mean spore length with standard deviation, W = mean spore width with standard deviation, Q = variation in L/W ratio, and n = number of spores measured from each specimen. Apiculi and ornamentation were excluded in spore measurements. Living mycelia were isolated from the woody substratum beneath the basidiocarps, and were cultured on 1.5% malt extract agar (MEA). Fungal specimens and living cultures used in this study are deposited in the herbaria of the National Museum of Natural Science of ROC (TNM; Taichung City, Taiwan) and Beijing Forestry University (BJFC; Beijing, China).

### DNA extraction, polymerase chain reaction (PCR), and sequencing

Dried specimens or the mycelial colonies cultured on MEA were used for DNA extraction, carried out with a Plant Genomic DNA Extraction Miniprep System (Viogene-Biotek Corp., New Taipei City, Taiwan). Liquid N and Tissue Lyser II (Qiagen, Hilden, Germany) were used to disrupt and homogenize the fungal tissues before DNA extraction process. The primer pairs ITS1/ITS4 or ITS1F/LR22 were used for the ITS region (White et al. 1990, Gardes and Bruns 1993), and LR0R/LR3 and LR0R/LR5 were used for the 28S region (Vilgalys and Hester 1990). Efdf/1953R and 983F/2218R were used to amplify a portion of the *TEF1* gene (Rehner & Buckley 2005; Matheny et al. 2007). PCR products were purified and directly sequenced by MB Mission Biotech Company (Taipei City, Taiwan). We examined the technical quality of the newly obtained sequences by comparison to entries in GenBank. Sequences were assembled using BioEdit v7.2.5 (Hall 1999). Newly obtained sequences (Supplementary Table 1) were submitted to either GenBank through the National Center for Biotechnology Information (NCBI) or DNA Data Bank of Japan (DDBJ) (Mashima et al. 2016, Benson et al. 2018).

### Alignment and phylogenetic analyses

The newly generated sequences were added to the DNA sequence dataset employed by Dai and He (2016), so far the most inclusive alignments for analyzing *Aleurodiscus* s.l. based on three genetic markers. To achieve a comprehensive analysis, we also added some related taxa of the genera *Boidinia* Stalpers & Hjortstam, *Conferticium* Hallenb., *Gloeocystidiellum* Donk and *Megalocystidium* Jülich to the ingroup. We tried to include the type species of the genera as far as possible (Table 1). The phylogenetic tree of the 28S+ITS+ *TEF1* dataset was inferred through Maximum likelihood (ML) and Bayesian inference (BI) methods using RAxML v. 8.2.4 (Stamatakis 2014) and MrBayes v. 3.2.6 (Ronquist et al. 2012), respectively (Ronquist et al. 2012, Stamatakis 2014). The alignments were inferred in MAFFT v. 7 using the FFT-N-i strategy for 28S and *TEF1*, and Q-INS-i strategy for ITS. For the BI analysis, the best-fit model for each alignment partition was estimated by jModelTest 2 (Darriba et al. 2012) using the Akaike information criterion (AIC). For ML bootstrapping, the extended majority-rule consensus tree criterion was specified under a GTRGAMMA model with 1000 replicates. In the BI analysis, four MCMC chains were run simultaneously from a random starting tree for ten million generations. Trees were sampled every 1000 generations resulting in 10000 trees in the posterior distribution; the first 25% trees were discarded as the burn-in. Posterior probabilities (PP) were calculated based on the post-burn-in trees. ML bootstrap values (BS) and BI posterior probability (PP) values ≥ 50% and ≥ 0.7 are indicated at the nodes of the ML tree. The final sequence alignments and the phylogenetic trees are available at TreeBASE (S23581; www.treebase.org).

**Table 1.** List of species, specimens and sequences used in this study. Sequences generated in this study are shown in boldface.

Fungal species	Specimen or strain no.	DDBJ/GenBank/EMBL accession no.		
		ITS	28S	*TEF1*
* Acanthobasidium bambusicola *	He2357	KU559343	KU574833	–
*Acanthofungusrimosus*#	Wu9601-1	MF043521	AY039333	–
* Acanthophysellum cerussatum *	He20120920-3	KU559339	KU574830	KU992716
*Aleurobotrysbotryosus*#	He2712	KX306877	KY450788	–
* Aleurocystidiellum disciforme *	He3159	KU559340	KU574831	KU992721
*Aleurocystidiellumsubcruentatum*#	He2886	KU559341	KU574847	KU992720
*** Aleurodiscus alpinus ***	**Wu1407-59**	**MF043522**	**MF043527**	–
*** Aleurodiscus alpinus ***	Wu1407-55*	–	**MF043526**	**LC269190**
*** Aleurodiscus alpinus ***	**Wu1407-61**	**MF043523**	**MF043528**	–
*Aleurodiscusamorphus*#	Ghobad-Nejhad-2464	KU559342	KU574832	KU992717
*Aleurodiscusamorphus*#	KHL4240	AF506397	AF506397	–
* Aleurodiscus bambusinus *	He 4261	KY706207	KY706219	**LC430911**
* Aleurodiscus canadensis *	Wu 1207-90	KY706203	KY706225	–
* Aleurodiscus dextrinoideocerussatus *	EL25-97	AF506401	AF506401	–
* Aleurodiscus dextrinoideophyses *	He 4105	MH109050	KY450784	–
* Aleurodiscus effusus *	He2261	KU559344	KU574834	KU992719
* Aleurodiscus gigasporus *	Wu 0108-15	KY706205	KY706213	–
* Aleurodiscus grantii *	HHB-14417	KU559363	KU574821	KU992708
* Aleurodiscus grantii *	HHB-14418	KU559364	KU574822	–
* Aleurodiscus isabellinus *	He 5283	MH109052	MH109046	**LC430912**
* Aleurodiscus mesaverdense *	FP-120155	KU559359	KU574817	–
* Aleurodiscus mirabilis *	Dai13281	KU559350	KU574839	KU992711
* Aleurodiscus oakesii *	He2243	KU559352	KU574840	–
* Aleurodiscus oakesii *	HHB11890-A-sp	KU559365	KU574823	–
*** Aleurodiscus pinicola ***	**Wu1106-16**	**MF043524**	**MF043529**	–
*** Aleurodiscus pinicola ***	Wu1308-54*	**MF043525**	**MF043530**	**LC269191**
*** Aleurodiscus senticosus ***	Wu1209-7*	**MH596849**	**MF043531**	**LC271169**
*** Aleurodiscus senticosus ***	**Wu1209-9**	**MH596850**	**MF043533**	**LC269192**
*** Aleurodiscus senticosus ***	**Wu9610-1**	**MH596851**	**MF043532**	**LC269193**
*** Aleurodiscus sichuanensis ***	Wu0010-18*	**MH596852**	**MF043534**	**LC269194**
*** Aleurodiscus sichuanensis ***	He 4935	**LC430904**	**LC430907**	–
* Aleurodiscus subroseus *	He 4807	MH109054	MH109048	–
*** Aleurodiscus subroseus ***	He 4895	**LC430903**	**LC430910**	**LC430913**
* Aleurodiscus tenuissimus *	He3575	KX306880	KX842529	–
* Aleurodiscus thailandicus *	He 4099	KY450781	KY450782	–
* Aleurodiscus tropicus *	He3830	KX553875	KX578720	LC269195
* Aleurodiscus verrucosporus *	He 4491	KY450786	KY450790	–
* Aleurodiscus wakefieldiae *	He2580	KU559353	KU574841	KU992710
* Boidinia macrospora *	Wu9202-21	AF506377	AF506377	–
* Conferticium heimii *	CBS321.66	AF506381	AF506381	–
* Conferticium ravum *	NH13291	AF506382	AF506382	–
* Gloeocystidiellum aspellum *	LIN625	AF506432	AF506432	–
***Gloeocystidiellumporosum***#	**Wu 1608-176**	**LC430905**	**LC430908**	–
* Gloeocystidiopsis cryptacanthus *	KHL10334	AF506442	AF506442	–
*Gloeocystidiopsisflammea*#	CBS324.66	AF506437	AF506437	–
* Heterobasidion parviporum *	91605	KJ651503	KJ651561	KU985089
* Megalocystidium chelidonium *	LodgeSJ110.1	AF506441	AF506441	–
*Megalocystidiumleucoxanthum*#	HK9808	AF506420	AF506420	–
* Megalocystidium wakullum *	Oslo-930107	AF506443	AF506443	–
*Neoaleurodiscusfujii*#	He2921	KU559357	KU574845	KU992709
* Stereum complicatum *	He2234	KU559368	KU574828	KU992706
*Stereumhirsutum*#	Wu 1109-127	**LC430906**	**LC430909**	–
* Stereum ostrea *	SHe2067	KU559366	KU574826	KU992703
* Stereum sanguinolentum *	He2111	KU559367	KU574827	KU992705
* Xylobolus frustulatus *	He2231	KU881905	KU574825	KU992704

* Holotype, # Generic type

## Phylogeny results

**Figure 1.**
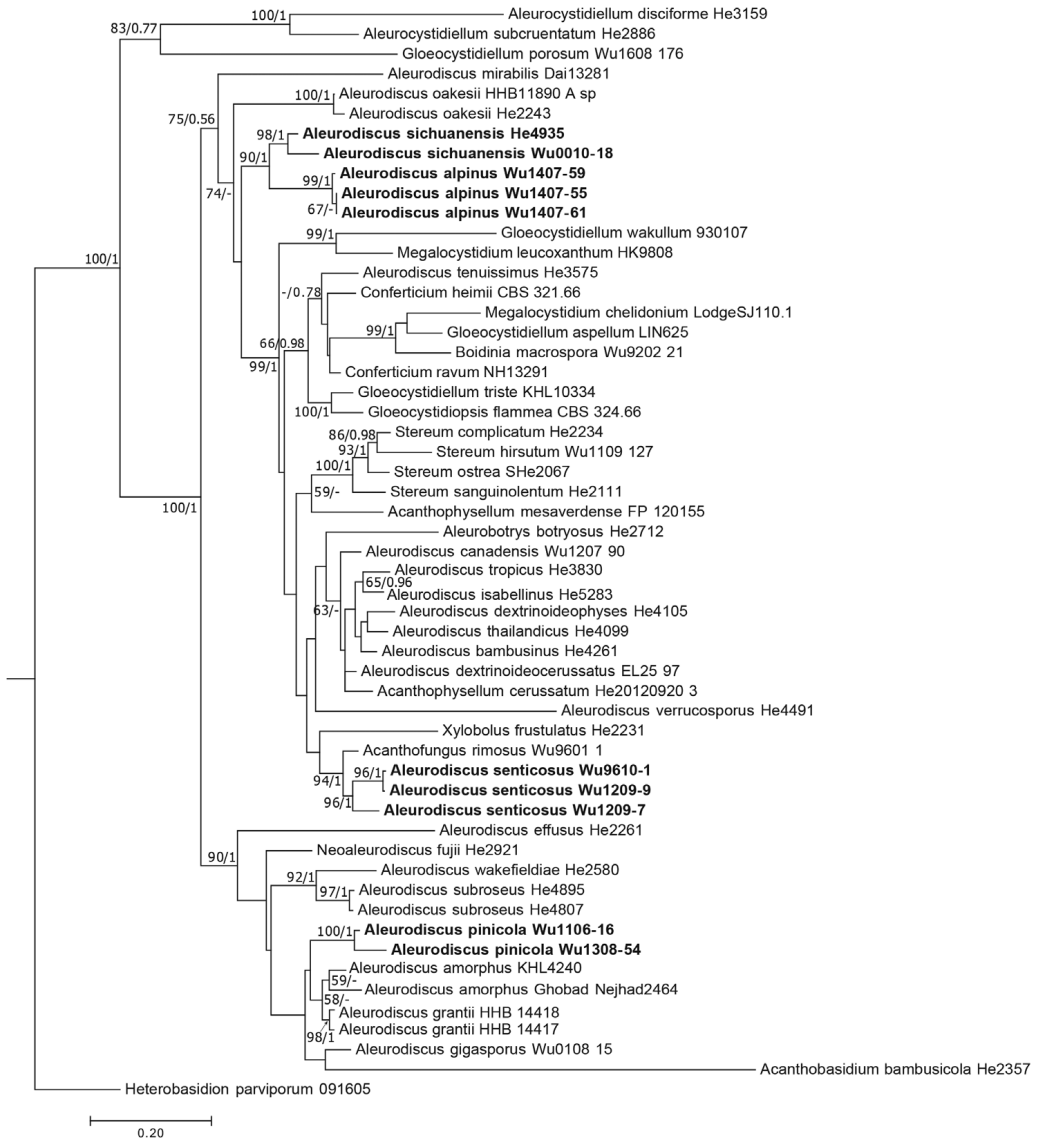
ML tree of *Aleurodiscus* and related genera of Stereaceae inferred from the 28S-ITS-*TEF1* markers. ML bootstrap values ≥ 50% and PP ≥ 0.7 from the Bayesian analysis are indicated at internodes. The presented new species are shown in boldface type.

The three-marker dataset was composed of 55 taxa and 2502 sites including gaps (of which 29% were parsimony-informative): 953 characters for 28S, 949 characters for ITS and 600 characters for *TEF1*. Missing sequences were treated as missing data (Table 1). After the ML search, 1000 rapid bootstrap inferences were executed. For the BI analysis, the GTR+I+G model was chosen as the best model for the 28S and *TEF1* alignments, and GTR+G was chosen for the ITS alignment. After 2.79 million generations, average standard deviation of split frequencies fell to 0.0099. Only the ML tree is shown given that the ML and BI analyses yielded similar topologies. The ML tree of the combined 28S, ITS and *TEF1-α* dataset (Fig. 1) showed that strains of *Aleurodiscusalpinus*, *A.pinicola*, *A.sichuanensis*, and *A.senticosus* formed separate clades in distinct lineages with high statistical support (BS = 96–100%, PP = 1). The strain of *A.sichuanensis* was sister to *A.alpinus* with significant support, BS: 90% and PP: 1.

## Taxonomy

### Aleurodiscus alpinus

Taxon classificationFungiRussulalesStereaceae

Sheng H. Wu sp. nov.

823178

Figs 2A , 3

#### Typification.

CHINA. YUNNAN PROVINCE: Shangrila County, Pudacuo National Park, Bita Lake, 27°43'N, 99°58'E, 3640 m, on branch of *Rhododendron* sp., 10 Jul 2014, *S.H. Wu*, *Wu 1407-55* (holotype TNM F27976). GenBank 28S = MF043526, *TEF1* = LC269190.

#### Etymology.

*alpinus* (L.), referring to the occurrence at high elevations.

#### Diagnosis.

Resembles *Aleurodiscuscupulatus* Núñez & Ryvarden in having discoid basidiomes, clamped hyphae, similar gloeocystidia, absence of acanthophyses, branched or unbranched hyphidia, and echinulate basidiospores. *Aleurodiscuscupulatus* features much wider basidiospores than *A.alpinus*. It differs from its closest phylogenetic relative, *A.sichuanensis*, by having clamped hyphae, but lacks acanthophyses.

#### Description.

Basidiomes cupuloid or discoid, solitary, occasionally fused, adnate, 350–750 μm thick in section. Hymenial surface Buff, Pale Luteous or Luteous, subceraceous, covered with crystal masses, not cracked; margin concolorous or paler, incurved, filamentous.

Hyphal system monomitic; hyphae nodose-septate. Pileus hyphae subcolorless to brownish, straight, thick-walled, walls usually thinner towards apices, usually with excreted material near apices. Subiculum uniform, with dense to compact texture, 150–500 μm thick; hyphae near substrate more or less vertical, moderately ramified, colorless, 3.5–8 μm diam, with 0.7–1.5 μm thick walls, occasionally guttulate; hyphae near hymenial layer more or less vertical, moderately ramified, colorless, fairly straight, 2.5–5 μm diam, thin- or slightly thick-walled, anastomoses occasional. Hymenial layer thickening, subhymenium differentiated from subiculum, 200–250 μm thick, with dense texture; hyphae fairly vertical, colorless, guttulate, 2–4 μm diam, thin-walled. Crystals sparsely scattered throughout section. Gloeocystidia numerous, immersed or slightly projecting, tubular, sometimes with adventitious septa near basal parts, colorless, (50–)70–200 × 4.5–12.5 μm, thin-walled, guttulate, SA+. Hyphidia numerous, sometimes branched, 40–130 × 2–6.5 μm. Basidia narrowly clavate, occasionally with one or two small protuberances, 85–165 × 16–20 μm, slightly thick-walled (ca. 0.5 μm thick), 4-sterigmate. Basidiospores ellipsoid to narrowly ellipsoid, adaxially concave, finely aculeate, thin-walled, homogenous or guttulate, amyloid, CB–, mostly 22–26 × 11–14 μm. (22–)22.2–26(–27.8) × (11–)11.8–13.5(–14.8) μm, L = 24.2±1.7 μm, W = 12.6±2.2 μm, Q = 1.95 (n = 30) (holotype, *Wu 1407-55*); (22–)23–24.5(–26) × (10.2–)10.8–13(–14) μm, L = 23.8±1.0 μm, W = 11.8±1.0 μm, Q = 2.02 (n = 30) (*Wu 1407-59*).

**Figure 2.**
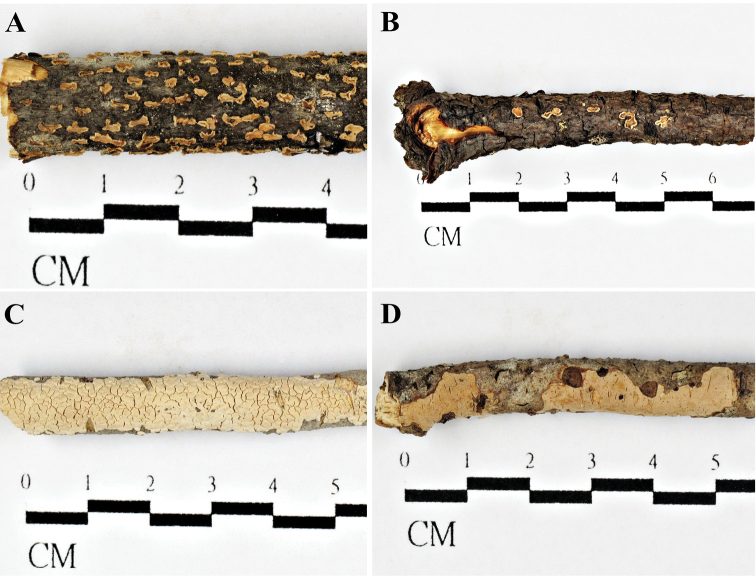
Basidiocarps **A***Aleurodiscusalpinus* (holotype, *Wu 1407-55*) **B***A.pinicola* (holotype, *Wu 1308-54*) **C***A.senticosus* (holotype, *Wu 1308-54*) **D***A.sichuanensis* (holotype, *Wu 0010-18*).

#### Ecology and distribution.

On dead branches of *Rhododendron* and other angiosperms at very high elevations, China, Jul.

#### Additional specimens examined.

CHINA. YUNNAN PROVINCE: Shangrila County, Pudacuo National Park, Bita Lake, 27°43'N, 99°58'E, 3640 m, on branch of *Rhododendron* sp., 10 Jul 2014, *S.H. Wu*, *Wu 1407-59* (TNM F27979), *Wu 1407-61* (TNMF27981); Pudacuo National Park, 3600 m, on dead branch of *Rhododendron* sp., 28 Jul 2017, *S.H. He*, *He 4924* (BJFC), *He 4942* (BJFC); Jianchuan County, Laochunshan, 26°38'N, 99°47'E, 3400 m, on angiosperm branch, 26 Jul 2001, *S.H. Wu* & *S.Z. Chen*, *Wu 0107-22* (TNMF13507), *Wu 0107-25* (TNMF13510).

**Figure 3.**
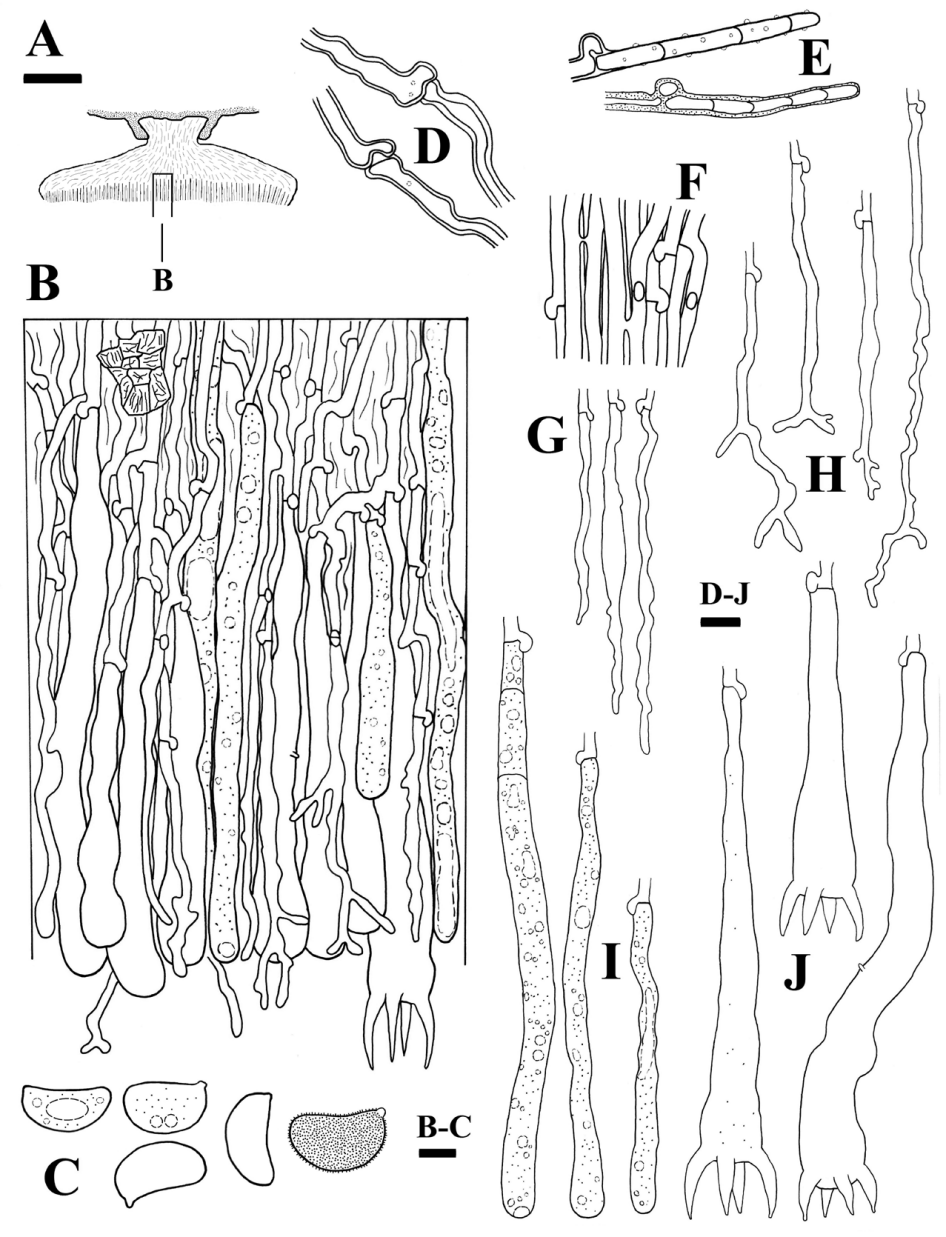
Microscopic structures of *Aleurodiscusalpinus* (holotype, *Wu 1407-55*) **A** profile of basidiocarp section **B** subhymenial and hymenial section **C** basidiospores (far right: in IKI) **D** subicular hyphae near substrate **E** pileus hyphae **F** subhymenial hyphae **G** hyphidia **H** branched hyphidia **I** gloeocystidia **J** basidia. Bars: 300 μm (**A**); 10 μm (**B–J**).

### Aleurodiscus pinicola

Taxon classificationFungiRussulalesStereaceae

Sheng H. Wu sp. nov.

823179

Figs 2B , 4

#### Typification.

CHINA. YUNNAN PROVINCE: Lichiang, High Mountain Workstation of Kunning Botanical Garden, 27°00'N, 100°11'E, 3250 m, on branch of *Pinusdensata*, 30 Aug 2013, *S.H. Wu*, *Wu 1308*-*54* (holotype TNM F27182). GenBank: ITS = MF043525, 28S = MF043530, *TEF1* = LC269191.

#### Etymology.

*pinicola* (L.), dwelling on *Pinus*, in reference to the substrate.

#### Diagnosis.

*Aleurodiscuspinicola* and *Acanthobasidiumpenicillatus* Burt share the features of moniliform gloeocystidia, acanthophyses with apical spines, dendrohyphidia, basidia with lateral protuberances, and aculeate basidiospores; the latter, however, has clamped hyphae and narrower basidiospores 18–27 × 12–14 (–17) μm. *Aleurodiscuspinicola* also resembles *A.oakesii* (Berk. & M.A. Curtis) Pat., however, the latter occurs on deciduous trees and has smaller basidiospores (15–20 × 13–17 μm).

Basidiomes discoid, each one up to 3.5 × 3 mm, adnate, membranaceous-subceraceous, 180–400 μm thick in section. Hymenial surface Buff or Pale Luteous, smooth, occasionally cracked; margin whitish, incurved, filamentous.

Hyphal system monomitic; most hyphae simple-septate, a few hyphal septa in junction of hymenium and subiculum with clamp connections. Subiculum uniform, with fairly dense dense texture, 60–160 μm thick; hyphae more or less vertical at resupinate parts, ± horizontal at marginal curved parts, moderately ramified, more or less interwoven, colorless, 2.5–6 μm diam, slightly thick-walled or containing thick walls up to 2 μm thick, sometimes with small oily drops, anastomoses occasional, some basal hyphae brownish yellow, with thicker walls than those elsewhere. Hymenial layer thickening, subhymenium more or less differentiated from subiculum, with dense texture, 100–270 μm thick; hyphae more or less vertical, colorless, sometimes with a short branch, usually containing minute oily drops, 2.5–5.5 μm diam, thin-walled. Crystal masses scattered throughout hymenial layer. Gloeocystidia numerous, mostly immersed or slightly projecting, cylindrical, usually strongly moniliform toward apices, usually forked, with numerous minute oily drops, colorless, 65–200 × 8.2–15.5 μm, thin- to slightly thick-walled, SA–. Acanthophyses numerous, clavate to broadly clavate, fusiform, stalked, colorless, apical parts with numerous protuberances, 50–100 × 5–30 μm, up to 1.2 μm thick walls, aculei 1–7 × 1–2 μm. Dendrohyphidia numerous, 37–90 × 3–4.8 μm. Hyphidia numerous, 35–80 × 2.4–4.2 μm. Basidia clavate, middle parts usually with several protuberances, 65–130 × 20–32 μm, up to 1.2 μm thick walls, 4-sterigmate. Basidiospores broadly ellipsoid to subglobose, adaxially flattened, aculeate, thin- to thick-walled, up to 3 μm thick walls, with a distinct apiculus, homogenous or with several oil-drops, amyloid, CB–, mostly 22.5–27.5 × 19–24 μm. (22.5–)23.5–27.2(–29) × (18.2–)19.2–22.8(–24) μm, L = 25.4±1.3 μm, W = 20.7±1.6 μm, Q = 1.23 (n = 30) (holotype, *Wu 1308-54*); (22.2–)23–26.5(–28) × (18.2–)20–22.5(–25.5) μm, L = 24.8±1.3 μm, W = 21.2±1.5 μm, Q = 1.17 (n = 30) (*Wu 1106-14*).

**Figure 4.**
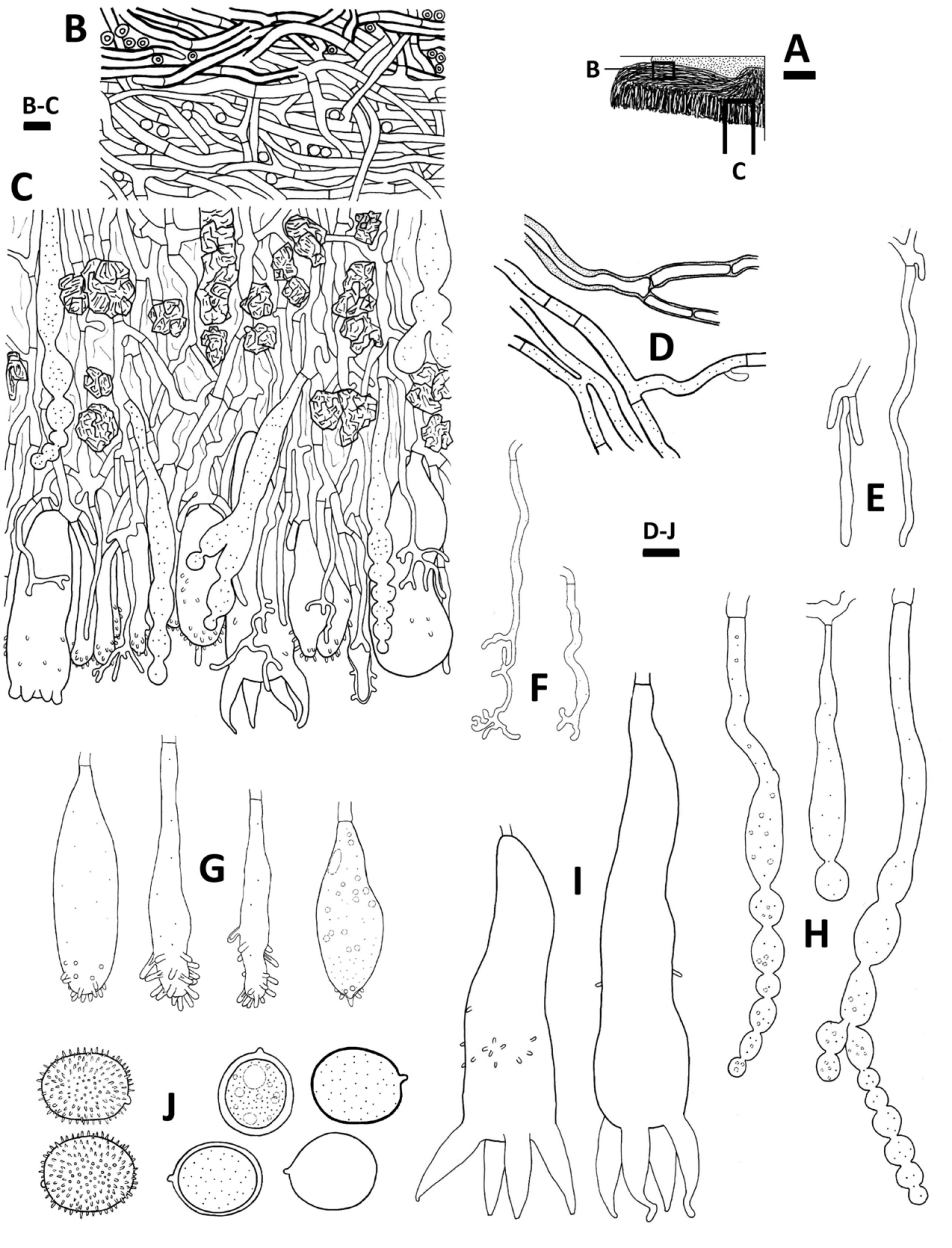
Microscopic structures of *Aleurodiscuspinicola* (holotype, *Wu 1308*-*54*) **A** profile of basidiocarp section **B** subicular hyphae of basidiocarp section **C** subhymenial and hymenial section **D** generative hyphae **E** hyphidia **F** dendrohyphidia **G** acanthophyses **H** gloeocystidia **I** basidia **J** basidiospores (left: in IKI, right: in KOH). Scale bars: 200 μm (**A**); 10 μm (**B–J**).

#### Ecology and distribution.

On *Pinus* branches at high elevations, China and Taiwan, Jun to Aug.

#### Additional specimens examined.

TAIWAN. Taichung, Siaosyueshan, Tienchih, 24°17'N, 121°01'E, 2580 m, on branch of *Pinusarmandii*, 8 Jun 2011, *S.H. Wu*, *Wu 1106-14* (TNM F25532); *ibid*. *Wu 1106-16* (TNMF25534).

### Aleurodiscus senticosus

Taxon classificationFungiRussulalesStereaceae

Sheng H. Wu sp. nov.

823180

Figs 2C , 5

#### Typification.

TAIWAN. New Taipei City, Wulai, 24°51'N, 121°33'E, 448 m, on branch of angiosperm, 10 Sep 2012, *S.H. Wu*, *Wu 1209-7* (holotype TNM F26702). GenBank: ITS = MH596849, 28S = MF043531, *TEF1* = LC271169.

#### Etymology.

*senticosus* (L.) = full of thorns, referring to the surface of basidia and cystidia.

#### Diagnosis.

Macroscopically featured in having a more or less cracked hymenophore, resulting from the fusion of numerous basidiome patches. Microscopically its basidia are diagnostic in having large lateral echinulate bladder-like swollen structure. Morphologically it resembles *Xylobolus* spp., although the latter cause a white-pocket rot in wood and have smooth basidiospores.

#### Description.

Basidiomes resupinate, beginning as small orbicular patches, gradually extending and fusing together then becoming effused, adnate, membranaceous, 250–600 μm thick in section. Hymenial surface Buff or Light Buff, slightly tuberculate, with a more or less cracked hymenophore; margin paler, usually determinate, occasionally thinning and byssoid.

Hyphal system monomitic; hyphae simple-septate, colorless. Subiculum with dense texture, 200–350 μm thick; hyphae next to substrate more or less horizontal, slightly interwoven, colorless, moderately ramified, at the junction of basidiocarp patches more or less vertical, 2–4(–5) μm diam, walls up to 1.5 μm thick. Hymenial layer thickening, with dense texture, 150–250 μm thick, not clearly differentiated from the subiculum; hyphae mainly vertical, colorless, 2–4 μm diam, thin- to slightly thick-walled. Gloeocystidia numerous, immersed or slightly projecting, cylindrical or tubular, with stalked bases, apically sometimes forked, sometimes with one or more constrictions near apices or slightly moniliform, colorless, 45–135 × 5–12 μm, with walls up to 1.5 μm thick, SA–. Acanthophyses numerous, subclavate or clavate, basal parts thin-walled, thick-walled toward apices, colorless, median to apical parts echinulate, 25–65 × 4–13 μm (spines excluded). Hyphidia numerous, 35–65 × 2–4 μm. Basidia clavate, 60–82 × 10–15 μm, with walls up to 2 μm thick, 4-sterigmate, usually with large lateral echinulate bladder-like swollen structure. Basidiospores broadly ellipsoid to subglobose, adaxially flattened, aculeate, with 1–3 μm thick walls, homogeneous or sometimes with several oily drops, amyloid, CB–, mostly 13.5–16.5 × 11–13 μm. (13–)13.5–15.8(–17) × (10–)11.2–12.5(–13) μm, L = 14.8±1.00 μm, W = 11.8±0.6 μm, Q = 1.25 (n = 30) (holotype, *Wu 1209-7*); (13–)14–16(–17.2) × (10–)11.2–13(–15) μm, L = 15.1±1.0 μm, W = 11.9±1.0 μm, Q = 1.26 (n = 30) (*GC 1604-46*).

**Figure 5.**
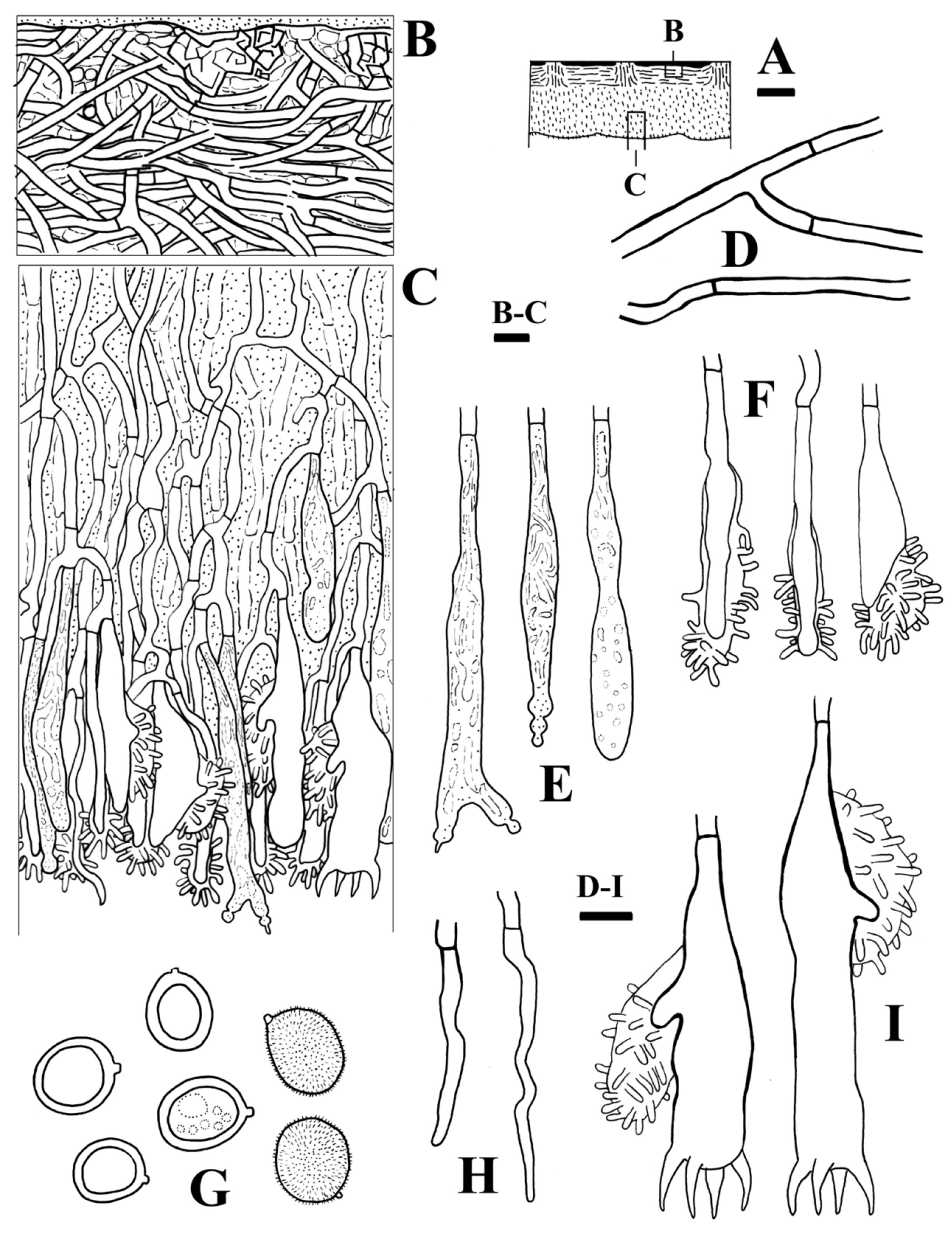
Microscopic structures of *Aleurodiscussenticosus* (holotype, *Wu 1209-7*) **A** profile of basidiocarp section **B** basal of basidiocarp section **C** section of hymenium **D** subicular hyphae **E** gloeocystidia **F** acanthophyses **G** basidiospores (left: in KOH, right: in IKI) **H** hyphidia **I** basidia. Scale bars: 200 μm (**A**); 10 μm (**B–I**).

#### Ecology and distribution.

On angiosperm branches, Taiwan, Apr to Sep.

#### Additional specimens examined.

Taiwan, New Taipei City, Wulai, 24°51'N, 121°33'E, 448 m, on angiosperm branch, 10 Sep 2012, *S.H. Wu*, *Wu 1209-9* (TNM F26704); Nantou, Lienhuachih. 23°56'N, 120°53'E, 700 m, on angiosperm branch, 08 Oct 1996, *S.H. Wu*, *Wu 9610-1* (TNM F5344); on angiosperm branch, 09 Apr 2016, *G.C. Chen*, *GC 1604-46* (TNM F30771).

### Aleurodiscus sichuanensis

Taxon classificationFungiRussulalesStereaceae

Sheng H. Wu sp. nov.

823181

Figs 2D , 6

#### Typification.

CHINA. SICHUAN PROVINCE: Wolungshan, Tengsheng, 2700 m, under bark of angiosperm, 11 Oct 2000, *S.H. Wu & S.C. Wu*, *Wu 0010-18* (holotype TNM F12097). GenBank: ITS = MH596852, 28S = MF043534, *TEF1* = LC269194.

#### Etymology.

*sichuanensis* (L.), referring to Sichuan Province, the type locality.

#### Diagnosis.

*Aleurodiscussichuanensis* resembles *A.oakesii* in having acanthophyses, simple-septate generative hyphae, and gloeocystidia occasionally with protuberances. However, clamped hyphae are rarely present in *A.oakesii*. Protuberances of acanthophyses of *A.oakesii* are antler-like, while aculei of acanthophyses in *A.sichuanensis* are fairly small. Basidiospores of *A.sichuanensis* are D-shaped or broadly ellipsoid, while those of *A.oakesii* are ovoid-ellipsoid and slightly smaller (18–27 ×12–14(–17) μm). *Aleurodiscussichuanensis*, however, is most closely related to *A.alpinus* and differs from it by having acanthophyses and simple-septate hyphae.

Basidiomes resupinate, effused, adnate, membranaceous-subceraceous, 150–350 μm thick in section. Hymenial surface smooth, Buff or Buff Yellow, occasionally cracked; margin concolorous, determinate.

Hyphal system monomitic; hyphae simple-septate. Subiculum uniform, with dense texture, thin or up to 150 μm thick; hyphae interwoven, colorless, richly ramified, tortuous, usually full of small oily drops, 2.5–5.5 μm diam, thin-walled. Hymenial layer with dense texture, 100–200 μm thick; hyphae vertical, colorless, ± straight, 2.5–4.5 μm diam, thin-walled. Crystal masses scattered in subiculum, yellowish. Gloeocystidia numerous, immersed or projecting, yellowish or pale brownish yellow, cylindrical, narrowly clavate or tubular, with oily contents or homogeneous, SA+, basal or median portion occasionally with small aculei, 70–135 × 7–14 μm, with 0.5–1 μm thick walls. Acanthophyses numerous, irregularly cylindrical or narrowly clavate, sometimes subfusiform, colorless, apical parts with numerous aculei, 30–70 × 3–8(–12) μm (aculei excluded), thin-walled. Hyphidia numerous, occasionally branched, 35–85 × 2.5–4.5 μm. Basidia clavate, 4-sterigmate, 100–130 × 20–25 μm, with 0.8–1.2 μm thick walls. Basidiospores D-shaped or broadly ellipsoid, adaxially flattened, finely aculeate, thin-walled or 1–2 μm thick, sometimes with oily contents, amyloid, CB–, mostly 25.5–28.5 × 15–18 μm. (25–)26–28.2(–29) × (14.5–)15.2–17(–19) μm, L = 27.1±1.0 μm, W = 15.9±1.1 μm, Q = 1.71 (n = 30) (holotype, *Wu 0010-18*).

**Figure 6.**
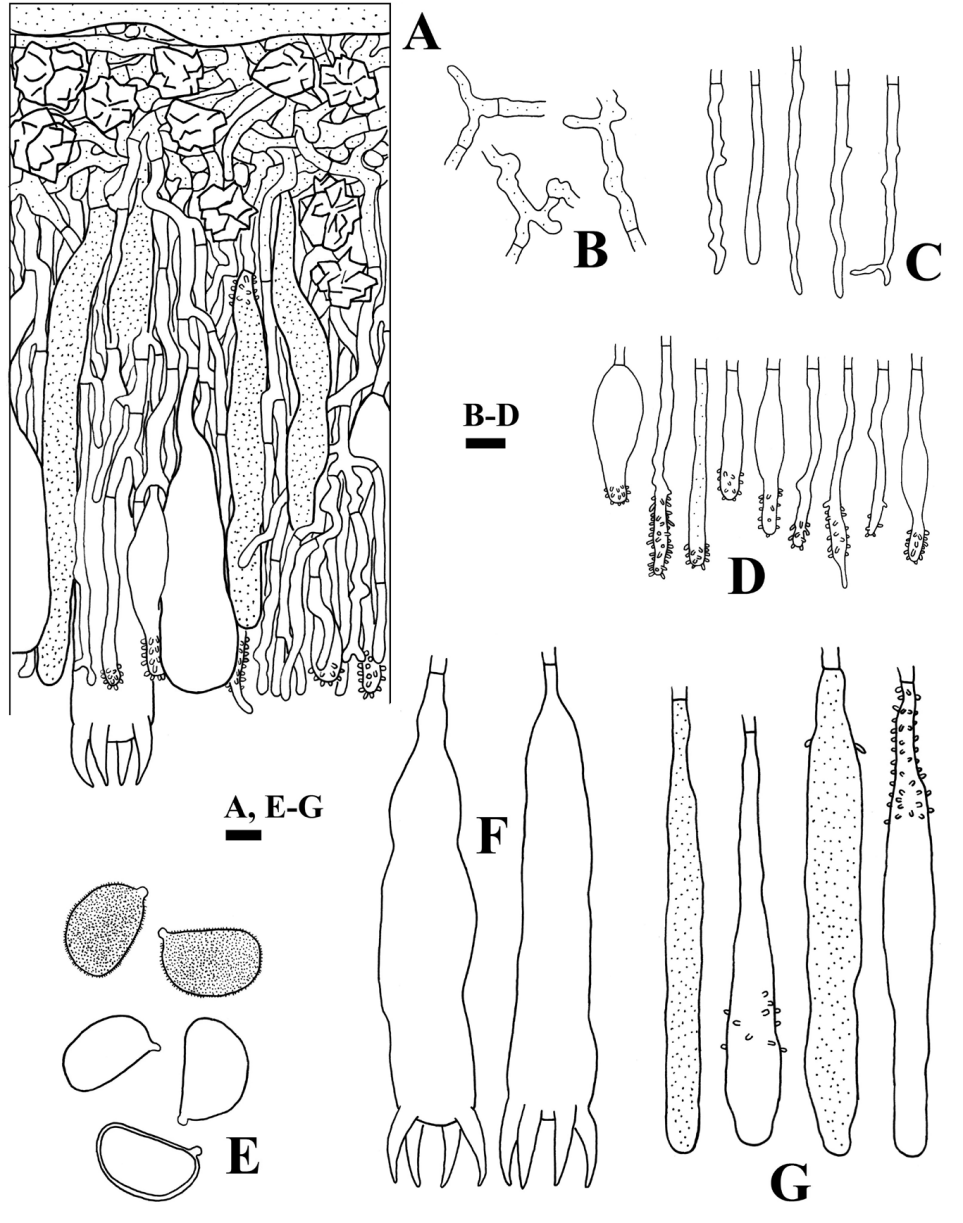
Microscopic structures of *Aleurodiscussichuanensis* (holotype, *Wu 0010-18*) **A** basidiocarp section **B** subicular hyphae **C** hyphidia **D** acanthophyses **E** basidiospores (upper: in IKI, lower: in KOH) **F** basidia **G** gloeocysitidia. Scale bars: 10 μm.

#### Ecology and distribution.

On dead branches of *Quercus* and other angiosperms at high elevations, China, Jul to Oct.

#### Additional specimens examined.

CHINA. SICHUAN PROVINCE: Wolungshan, Tengsheng, 2700 m, on angiosperm branch, 11 Oct 2000, *S.H. Wu & S.C. Wu*, *Wu 0010-42* (TNM F12118). YUNNAN PROVINCE: Shangrila County, Pudacuo National Park, 3600 m, on dead branch of *Quercusapuifolloides*, 28 Jul 2017, *S.H. He*, *He 4923*, *He 4926*, *He 4930*, *He 4935* (BJFC).

## Discussion

A number of phylogenetic studies of *Aleurodiscus* s.l. have been conducted in the past twenty years (Wu et al. 2001; Larsson and Larsson 2003; Miller et al. 2006; Larsson 2007; Dai and He 2016; Dai et al. 2017). Miller et al. (2006) and Larsson (2007) tried to establish a family level classification for *Aleurodiscus* s.l., as well as related taxa of the Russulales. However, a fully resolved and robust phylogeny of *Aleurodiscus* s.l. and related taxa was not achievable with ribosomal genes alone. Dai and He (2016) and our study have addressed this by including *TEF1* for phylogenetic analyses. From our phylogenetic analyses of three DNA genetic markers (Fig. 1) we can conclude the following about evolutionary relationships in the Stereaceae: (i) *Aleurodiscus* s.l. is highly polyphyletic; (ii) *Acanthophysellum* is polyphyletic; (iii) *Gloeocystidiellum* is polyphyletic; (iv) *Megalocystidium* is polyphyletic; and (v) *Conferticium* is paraphyletic.

*Aleurodiscusalpinus* is reminiscent of *Aleurodiscus* s.s. (*A.amorphus* (Pers.) J. Schröt. and *A.grantii* Lloyd) due to the discoid basidiocarp and echinulate basidiospores, as well as the absence of acanthophyses. However, the gloeocystidia of *Aleurodiscus* s.s. are paraphysis-like, narrow and moniliform, while those of *A.alpinus* are much wider and not moniliform. In addition, *A.alpinus* has unbranched or branched hyphidia, which are lacking in *Aleurodiscus* s.s. *Aleurodiscusalpinus* formed a clade with *A.sichuanensis* (Fig. 1), however, the latter has simple-septate hyphae and acanthophyses. *Aleurodiscusalpinus* and *A.cupulatus* share most morphological features, except the latter has much wider basidiospores. *Aleurodiscusalpinus* grows on *Rhododendron* sp. in Yunnan of China, while *A.cupulatus* occurs on *Pseudotsugamenziesii* in Idaho of USA. No DNA sequence of the latter has been obtained to examine their relationship.

*Aleurodiscuspinicola* presents protuberances in the basidia and this is reminiscent of *Acanthobasidium*. However, this feature is not limited to *Acanthobasidium* spp. For example, basidia of *Aleurodiscusmirabilis* (Berk. & M.A. Curtis) Höhn. and *A.wakefieldiae* Boidin & Beller occasionally possess protuberances, but they and *A.pinicola* do not belong to *Acanthobasidium* (Fig. 1).

*Aleurodiscussenticosus* is macroscopically distinct in having more or less cracked hymenophore from the fusion of smaller basidiocarp patches; microscopically, its basidia bear a large, spiny, bladder-like structure that is unique among *Aleurodiscus* s.l. The present phylogenetic analyses (Fig. 1) indicated that *A.senticosus* formed a clade with *Xylobolus* and *Acanthofungus*, but without strong support. However, these two genera differ from *A.senticosus* by causing a white-pocket rot in wood and by bearing smooth basidiospores.

*Aleurodiscussichuanensis* cannot be accommodated in any segregate genus of *Aleurodiscus* s.l., according to the combined features of effused basidiocarp, simple-septate hyphae, acanthophyses, gloeocystidia with aculei, and echinulate basidiospores.

In conclusion, the status of each segregate genus of *Aleurodiscus* s.l. should be further examined by multi-gene analysis of more species to evaluate which ones can be recognized and which cannot. Although the four new species we introduce cannot be accommodated in any segregate genus of *Aleurodiscus* s.l. according to the present combined morphological and phylogenetic studies, they are still placed under the broad sense of *Aleurodiscus* at the present time.

## Supplementary Material

XML Treatment for Aleurodiscus alpinus

XML Treatment for Aleurodiscus pinicola

XML Treatment for Aleurodiscus senticosus

XML Treatment for Aleurodiscus sichuanensis

## References

[B1] Benson DA Cavanaugh M Clark K Karsch-Mizrachi I Ostell J Pruitt KD Sayers EW (2018) GenBank. Nucleic Acids Research 46 (D1): D41–D46. 10.1093/nar/gkx1094 PMC5753231 29140468

[B2] Boidin J Lanquetin P Gilles G Candoussau F Hugueney R (1985) Contribution à la connaissance des Aleurodiscoideae à spores amyloides (Basidiomycotina, Corticiaceae). Bulletin de la Société Mycologique de France 101: 333–367.

[B3] Darriba D Taboada GL Doallo R Posada D (2012) jModelTest 2: more models, new heuristics and parallel computing. Nature Methods 9: 772–772. 10.1038/nmeth.2109 PMC4594756 22847109

[B4] Dai LD He SH (2016) New species and new records of *Aleurodiscus* s.l. (Basidiomycota) in China. Mycological Progress 15: 717–730. 10.1007/s11557-016-1202-z

[B5] Dai LD Wu SH Nakasone KK Burdsall HH He SH (2017a) Two new species of *Aleurodiscus* s.l. (Russulales, Basidiomycota) on bamboo from tropics. Mycoscience 58: 213–220. 10.1016/j.myc.2017.02.001

[B6] Dai LD Zhao Y He SH (2017b) Three new species of *Aleurodiscus* s.l. (Russulales, Basidiomycota) on bamboos from East Asia. Cryptogamie, Mycologie 38: 227–239. 10.7872/crym/v38.iss2.2017.227

[B7] Gardes M Bruns TD (1993) ITS primers with enhanced specificity for basidiomycetes - application to the identification of mycorrhizae and rusts. Molecular Ecology 2: 113–118. 10.1111/j.1365-294X.1993.tb00005.x 8180733

[B8] Ghobad-Nejhad M Langer E (2018) A new species in *Aleurodiscus* s.l. (Stereaceae, Russulales) from Iran. Phytotaxa 351: 264–272. 10.11646/phytotaxa.351.4.2

[B9] Gorjón SP Greslebin AG Rajchenberg M (2013) The genus *Aleurodiscus* s.l. (Stereaceae, Russulales) in the Patagonian Andes. Mycological Progress 12: 91–108. 10.1007/s11557-012-0820-3

[B10] Hall TA (1999) BioEdit: a user-friendly biological sequence alignment editor and analysis program for Windows 95/98/NT. Nucleic Acids Symposium Series 41: 95–98.

[B11] Hjortstam K Roberts PJ Spooner BM (2009) Corticioid fungi from the Kimberley region, Western Australia. Kew Bull 64: 353–368. 10.1007/s12225-009-9104-8

[B12] Larsson KH (2007) Re-thinking the classification of corticioid fungi. Mycological Research 111: 1040–1063. 10.1016/j.mycres.2007.08.001 17981020

[B13] Larsson E Larsson KH (2003) Phylogenetic relationships of russuloid basidiomycetes with emphasis on aphyllophoralean taxa. Mycologia 95: 1037–1065. 10.2307/3761912 21149013

[B14] Maninder K Avneet PS Dhingra GS Ryvarden L (2014) *Aleurodiscushimalaicus* (Agaricomycetes) sp. nov. from India. Synopsis Fungorum 32: 5–7.

[B15] Matheny BP Wang Z Binder M Curtis JM Lim YW Nilsson RH Hughes KW Hofstetter V Ammirati JF Schoch CL Langer E Langer G McLaughlin DJ Wilson AW Frøslev T Ge Z-W Kerrigan RW Slot JC Yang ZL Baroni TJ Fischer M Hosaka K Matsuura K Seidl MT Vauras J Hibbett DS (2007) Contributions of rpb2 and tef1 to the phylogeny of mushrooms and allies (Basidiomycota, Fungi). Molecular Phylogenetics and Evolution 43: 430–451. 10.1016/j.ympev.2006.08.024 17081773

[B16] Mashima J Kodama Y Kosuge T Fujisawa T Katayama T Nagasaki H Okuda Y Kaminuma E Ogasawara O Okubo K Nakamura Y Takagi T (2016) DNA data bank of Japan (DDBJ) progress report. Nucleic Acids Research 44: D51–D57. 10.1093/nar/gkv1105 PMC4702806 26578571

[B17] Miller SL Larsson E Larsson K-H Verbeken A Nuytinck J (2006) Perspectives in the new Russulales. Mycologia 986: 960–970. 10.1080/15572536.2006.11832625 17486972

[B18] Núñez M Ryvarden L (1997) The genus *Aleurodiscus* (Basidiomycotina). Synopsis Fungorum 12: 1–164.

[B19] Rayner RW (1970) A Mycological Colour Chart. Commonwealth Mycological Institute, Kew 34 pp.

[B20] Rehner SA Buckley E (2005) A *Beauveria* phylogeny inferred from nuclear ITS and EF1-α sequences: evidence for cryptic diversification and links to *Cordyceps* teleomorphs. Mycologia 97: 84–98. 10.1080/15572536.2006.11832842 16389960

[B21] Ronquist F Teslenko M Mark P van der Ayres DL Darling A Hohna S Larget B Liu L Suchard MA Huelsenbeck JP (2012) MrBayes 3.2: Efficient Bayesian phylogenetic inference and model choice across a large model space. Systematic Biology 61: 539–542. 10.1093/sysbio/sys029 22357727 PMC3329765

[B22] Ryvarden L Sanyal SK Dhingra GS (2012) *Aleurodiscusindicus* (Agaricomycetes) sp. nov. from India. Synopsis Fungorum 30: 14–16.

[B23] Simpson JA Grgurinovic CA (2003) A new species of *Aleurodiscus* (Stereaceae) from Mt Kosciuszko, Australia. Australasian Mycologist 22: 15–19.

[B24] Stamatakis A (2014) RAxML version 8: a tool for phylogenetic analysis and post-analysis of large phylogenies. Bioinformatics 30: 1312–1313. 10.1093/bioinformatics/btu033 24451623 PMC3998144

[B25] Tian Y Ghobad-Nejhad M He SH Dai YC (2018) Three new species of *Aleurodiscus* s.l. (Russulales, Basidiomycota) from southern China. MycoKeys 37: 93–107. 10.3897/mycokeys.37.25901 PMC6086924 30116140

[B26] Vilgalys R Hester M (1990) Rapid genetic identification and mapping of enzymatically amplified ribosomal DNA from several *Cryptococcus* species. Journal of Bacteriology 172: 4238–4246. 10.1128/jb.172.8.4238-4246.1990 2376561 PMC213247

[B27] White TJ Bruns TD Lee S Taylor J (1990) Amplification and direct sequencing of fungal ribosomal RNA genes for phylogenetics. In: Innis MA, Gelfand DH, Sninsky JJ, White TJ (eds) PCR protocols, a guide to methods and applications. Academic, San Diego, pp 315–322. 10.1016/B978-0-12-372180-8.50042-1

[B28] Wu SH Hibbett DS Binder M (2001) Phylogenetic analyses of *Aleurodiscus* s.l. and allied genera. Mycologia 93: 720–731. 10.2307/3761826

[B29] Wu SH Wang DM Yu SY (2010) *Neoaleurodiscusfujii*, a new genus and new species found at the timberline in Japan. Mycologia 102: 217–223. 10.3852/09-052 20120243

